# Supplementary material for: Stress Assessment of Vestibular Endurance Training for Civil Aviation Flight Students Based on EEG
Source: Front Hum Neurosci. 2021 Aug 19;15:582636. doi: 10.3389/fnhum.2021.582636 (PMC8417248; doi:10.3389/fnhum.2021.582636)
Supplement: Supplementary file 1 [file Data_Sheet_1.pdf]

Supplementary Material

1 SUPPLEMENTARY DATA

Table S1. Subjects' Data of C3 area

NO	b_all	b1	b5	a1	a2	a5	a_all	a2-b1	Ladder Performance (s)
NO. 2	35.36619643	31.65337231	36.65774777	45.7077	46.23617	50.79556	47.93719	14.5828	76
NO. 3	41.6294397	20.49946836	35.27005827	22.66018	27.27726	29.67471	27.45766	6.777794	58
NO. 4	38.28809673	12.46060639	56.77235637	42.6297	66.4411	64.2128	58.24283	53.98049	66
NO. 5	27.47808095	12.12898499	28.67899044	32.09288	32.98521	33.85611	33.35563	20.85623	64
NO. 6	63.59241142	44.95893076	62.0930518	125.8947	102.3806	97.72197	104.7701	57.42163	64
NO. 7	106.4749841	92.00932322	91.87221238	101.0825	132.6137	86.30664	102.5861	40.60442	68
NO. 9	72.08778188	66.65640202	71.79823695	79.74734	63.60383	84.36156	73.87828	-3.05257	73
NO. 10	19.93310768	9.449406737	23.98918338	23.72859	25.0145	25.35094	23.49712	15.5651	70

Table S2. Subjects' Data of C4 area

NO	b_all	b1	b5	a1	a2	a5	a_all	a2-b1	Ladder Performance (s)
NO.2	21.82557861	18.94438888	21.9120327	35.05500449	35.24350997	37.9636576	35.75600472	16.2991211	76
NO.3	57.6261363	29.98202681	53.63884994	26.53790465	31.50842288	28.97986293	28.72516697	1.526396074	58
NO.4	55.40431592	15.09449815	81.62878757	36.83754224	72.27865085	76.61668439	64.98001471	57.1841527	66
NO.5	30.01265579	11.06253546	32.06184949	33.87766744	37.01965201	35.68824946	36.37957223	25.95711655	64
NO.6	63.93710972	43.8917978	67.48119394	116.6155722	101.1429514	97.04217935	100.9284141	57.25115356	64
NO.7	101.9261412	85.74180869	93.19164327	92.0418994	122.4257649	85.93141537	96.10656226	36.6839562	68
NO.9	38.12305135	34.99862965	37.31265846	46.98535984	37.38603126	45.97968767	41.26257444	2.38740161	73
NO.10	29.08516759	14.53850856	33.33549247	31.44636756	32.4381904	31.04143948	31.13875225	17.89968184	70

2 SUPPLEMENTARY TABLES AND FIGURES

2.1 Figures

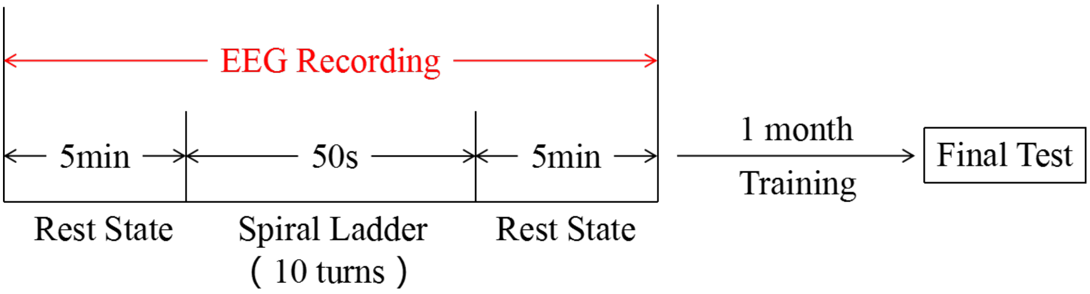

Figure S1. Experimental design

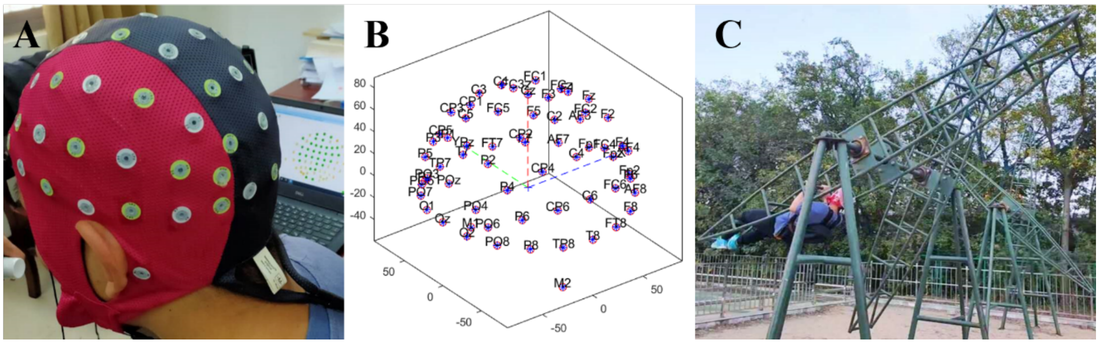

Figure S2. (A) showed the diagram of device wearing. (B) showed the location of channels. (C) showed the rotary ladder movement.

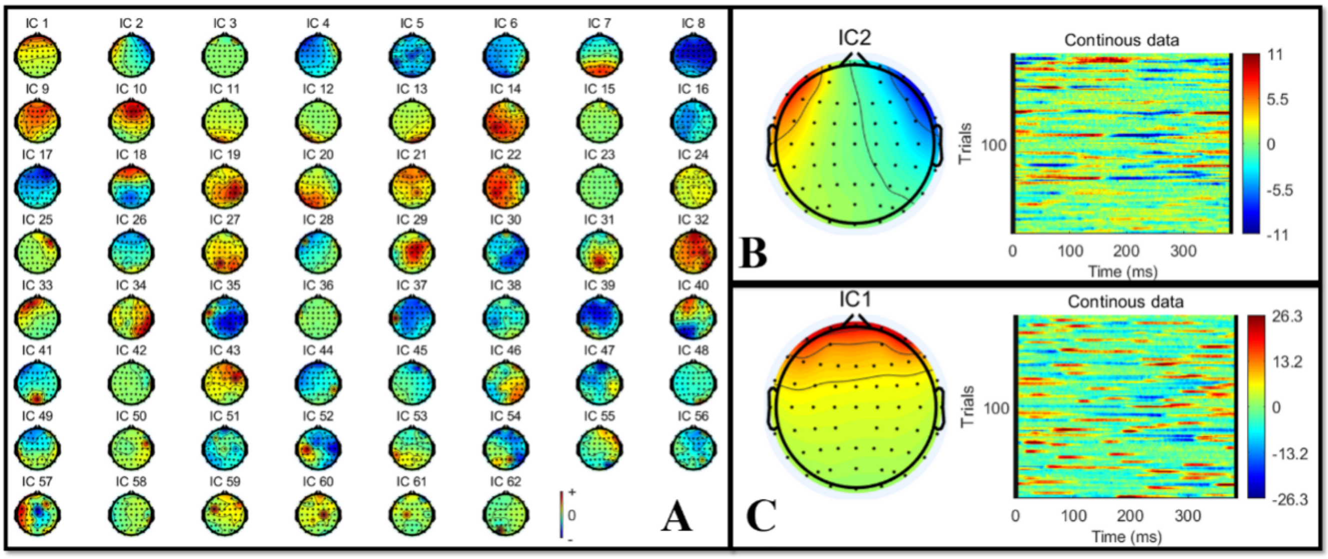

Figure S3. (A) showed the ICA component decomposition result. (B)-(C) showed the removed IC components' topographic map and time-frequency results. (B) showed the eye blinks activities component. (C) shows the lateral eye movements component.

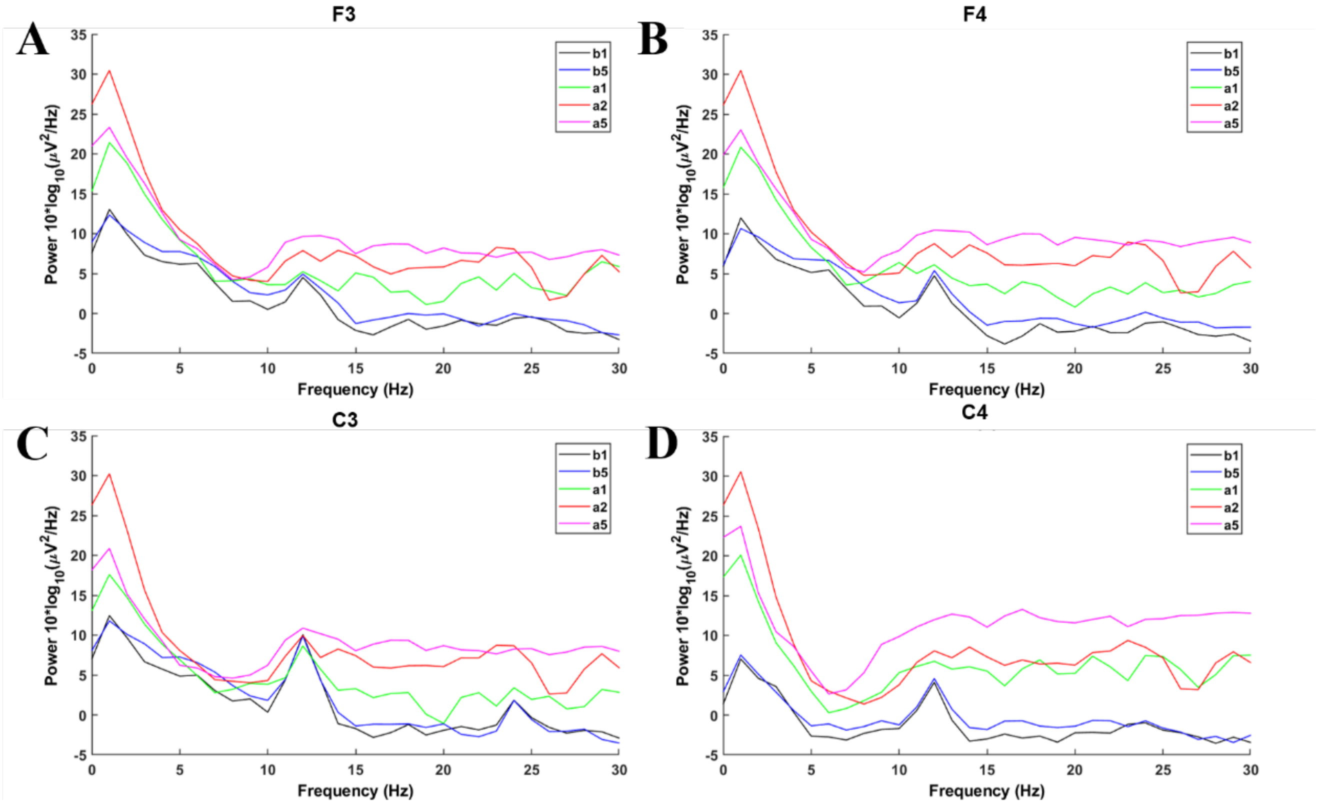

Figure S4. Power spectra. The power spectra of b_1 are shown as black lines, the power spectra of b_5 are shown as blue lines, the power spectra of a_1 are shown as green lines, the power spectra of a_2 are shown as red lines, the power spectra of a_5 are shown as pink lines. (A) shows the power spectra of F3 area, (B) shows the power spectra of F4 area, (C) shows the power spectra of C3 area, (D) shows the power spectra of C4 area.

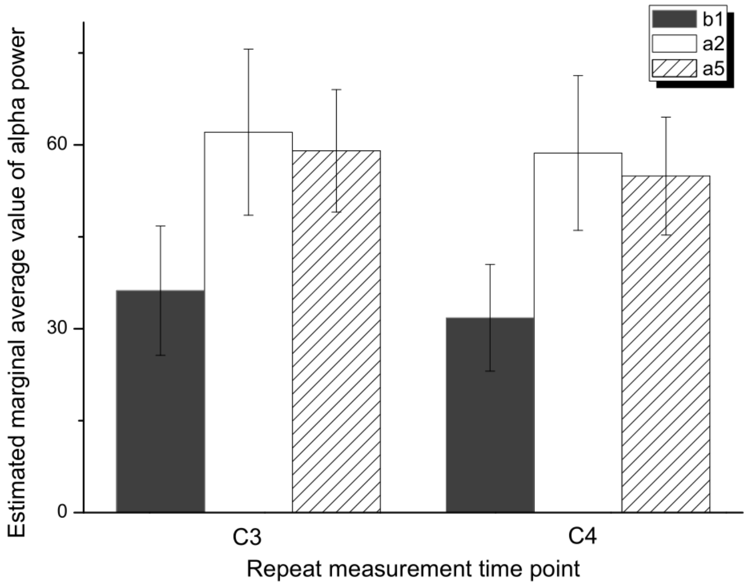

Figure S5. Variance analysis of repeated measurement of α absolute power in C3 and C4.

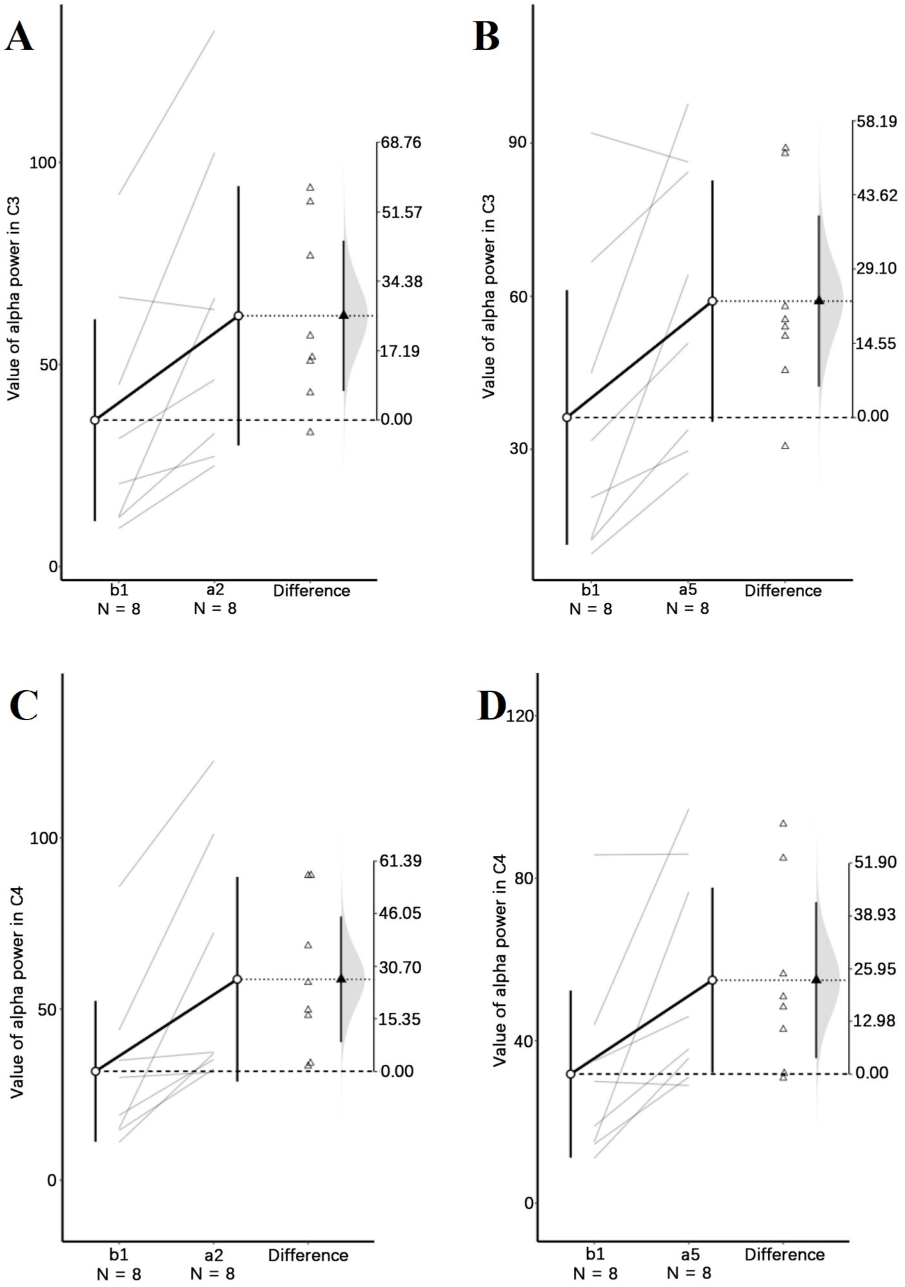

Figure S6. Visualizations emphasizing testing versus estimation. In this plot the lines represent the individual participants. The two ends of the line represent the pre- and post-stimulus values of the participant . The circles with error bars represent each group mean with their 95% confidence intervals. Critically, an estimation plot emphasizes the effect size of interest for this design: the difference between the group means. This is depicted on the "difference axis" on the right. The triangles show the difference between groups in this paired samples. The shaded curve shows the entire distribution of expected sampling error for the difference between the means. A,B,C,D respectively represents the descriptive plots of b1 and a2, b1 and a5 on the C3 channel, b1 and a2, b1 and a5 on the C4 channel.

Table S3. Correlation between expert's final performance and novice's EEG response of acute stimulation

	C3 (n=8)		C4 (n=8)	
	Mean (95% CI)	SE	Mean (95% CI)	SE
b_1	36.22 (11.25, 61.20)	10.56	31.78 (11.20, 52.36)	8.70
b_5	50.89 (31.06, 70.73)	8.39	52.57 (30.95, 74.19)	9.14
a_1	59.19 (26.95, 91.43)	13.63	52.42 (24.66, 80.18)	11.71
a_2	62.07 (30, 94.14)	13.56	58.68 (28.79, 88.27)	12.64
a_5	59.03 (35.37, 82.70)	10.00	54.91 (32.15, 77.66)	9.62

Table S4. EEG power spectrum of α band before and after the stimulation

Performance (s)	Power difference of α in C3 $X_1 = T_{a2} - T_{b1}$	Power difference of α in C4 $X_2 = T_{a2} - T_{b1}$	Spearman correlation coefficient with the performance after long-term training
76	14.58	16.3	$\rho_{X1} = -0.81$
66	53.98	57.18	$p = 0.027$
64	20.86	25.96	$\rho_{X2} = -0.81$
64	57.42	57.25	$p = 0.027$
68	40.60	36.68	(The performance of case 3 is 58s, which is
73	-3.05	2.39	much higher than the average value of
70	15.57	17.90	67.38s and rejected)
